# Boron-peptide conjugates with angiopep-2 for boron neutron capture therapy

**DOI:** 10.3389/fmed.2023.1199881

**Published:** 2023-06-01

**Authors:** Jing Xiang, Lin Ma, Jianfei Tong, Nan Zuo, Weitao Hu, Yupeng Luo, Junqi Liu, Tianjiao Liang, Qiushi Ren, Qi Liu

**Affiliations:** ^1^Institute of Biomedical Engineering, Peking University Shenzhen Graduate School, Shenzhen, China; ^2^Department of Stomatology, General Hospital, Shenzhen University, Shenzhen, Guangdong, China; ^3^Institute of High Energy Physics, Chinese Academy of Sciences (CAS), Beijing, China; ^4^Spallation Neutron Source Science Center, Dongguan, China; ^5^Institute of Biomedical Engineering, Shenzhen Bay Laboratory, Shenzhen, Guangdong, China; ^6^Department of Stomatology, The First Hospital, Harbin Medical University, Harbin, China; ^7^School of Stomatology, Hangzhou Normal University, Hangzhou, Zhejiang, China; ^8^School of Stomatology, Shenzhen University, Shenzhen, Guangdong, China; ^9^Department of Radiation Oncology, The First Affiliated Hospital of Zhengzhou University, Zhengzhou, China; ^10^International Cancer Center, Shenzhen University School of Medicine, Shenzhen University, Shenzhen, Guangdong, China

**Keywords:** Boron neutron capture therapy (BNCT), boron-peptide conjugates, angiopep-2, binary radiation therapy, cancer medicine

## Abstract

Boron neutron capture therapy (BNCT) induces intracellular nuclear reaction to destroy cancer cells during thermal neutron irradiation. To selectively eliminate cancer cells but avoid harmful effects on normal tissues, novel boron-peptide conjugates with angiopep-2, namely ANG-B, were constructed and evaluated in preclinical settings. Boron-peptide conjugates were synthesized using solid-phase peptide synthesis, and the molecular mass was validated by mass spectrometry afterwards. Boron concentrations in 6 cancer cell lines and an intracranial glioma mouse model after treatments were analyzed by inductively coupled plasma atomic emission spectroscopy (ICP-AES). Phenylalanine (BPA) was tested in parallel for comparison. *In vitro* treatment with boron delivery peptides significantly increased boron uptake in cancer cells. BNCT with 5 mM ANG-B caused 86.5% ± 5.3% of clonogenic cell death, while BPA at the same concentration caused 73.3% ± 6.0% clonogenic cell death. The *in vivo* effect of ANG-B in an intracranial glioma mouse model was evaluated by PET/CT imaging at 31 days after BNCT. The mouse glioma tumours in the ANG-B-treated group were shrunk by 62.9% on average, while the BPA-treated tumours shrank by only 23.0%. Therefore, ANG-B is an efficient boron delivery agent, which has low cytotoxicity and high tumour-to-blood ratio. Based on these experimental results, we expected that ANG-B may leverage BNCT performance in clinical applications in future.

## 1. Introduction

Boron neutron capture therapy utilizes the intracellular ^10^B (n,α) ^7^Li reaction resulted from boron-10 capture of thermal neutrons to destroy cancer cells. The heavy charged α and lithium (Li^3+^) particles produced by BNCT have very high linear energy transfer (LET) values (~230 keV/μm), generate densely ionizing events along the particle rays, give rise to highly complex and irreparable DNA damages, and result in robust cell kill (1, 2). Therefore, BNCT is supposedly a powerful way to manage cancers that are refractory to conventional therapy. BNCT has been tested in clinical trials to treat glioblastoma multiforme (GBM), melanoma, head and neck cancer, colon carcinoma liver metastases, etc. (3). Indeed, cyclotron-based BNCT with the boron delivery agent ^10^B-p-boronophenylalanine (BPA) was approved in 2020 for the treatment of head and neck cancer with health insurance coverage in Japan (4). Moreover, clinical trials on various cancer types suggested that BNCT may achieve better effects than conventional therapies in some instances (3, 5, 6).

The challenge of BNCT lies in developing stable and low-toxic boron carriers that can efficiently deliver ^10^B into tumour cells and stay long enough for ^10^B accumulation. The heavy charged particles from BNCT only travel for a cell length (~10 μm). In order to generate lethal DNA damages in the cell nucleus, boron delivery agents are usually developed with the capability of cell penetration. Furthermore, an effective BNCT requires boron concentration to reach 20 μg ^10^B per gram of tumour and tumour-to-normal tissue ratio (T/N ratio) to be above 3:1 by estimation (7). Thus, a qualified boron delivery agent should be able to concentrate ^10^B in cancer cells. BPA and sodium borocaptate (BSH) have been commonly used boron delivery agents in BNCT for decades (8). BPA has a chemical structure similar to tyrosine, so cancer cells take up a lot of BPA due to their enhanced need for amino acids. BSH carries 12 times more boron atoms per molecule than BPA but lacks molecular targeting effects. At the same time, many research groups are in pursuit of novel boron delivery agents with better performance in tumour targeting and cancer cell penetration (3, 6).

Physiological barriers prevent many active molecules from entering cancer cells or reaching a tumour, thus greatly restricting the application of boron delivery agents in BNCT (6, 9). For example, the presence of blood–brain barrier (BBB) is the fundamental problem impeding the development of new therapeutics for brain disease (10). As a gate that only allows transportation of particular substances between cytoplasm and the external environment, cell membrane is another barrier to drug development. To overcome these barriers, cell-penetrating peptides (CPPs) have received widespread interest in the field of drug development due to their advantages in intracellular transduction efficiency, chemical conjugation diversity, biocompatibility, and low cytotoxicity (11, 12). However, only a few peptides have been tested for boron delivery in BNCT (13, 14).

Here we aimed to develop novel boron delivery agents using the boron-peptide conjugates strategy. Angiopep-2 is a low-density lipoprotein that can specifically bind to the LRP-1 receptor which is highly expressed in the BBB and glioma cells. Therefore, angiopep-2 has been used to direct therapeutic and diagnostic agents to the brain in a number of studies (15), suggesting a boron-10 carrier based on angiopep-2 may be useful for BNCT of glioma as well as other cancer types. We designed and synthesized the N-terminal boron-containing derivative of angiopep-2, namely ANG-B, and assessed its effects as boron delivery agents for BNCT. Results from *in vitro* and *in vivo* experiments suggested that ANG-B has high boron delivery efficiency, low normal tissue cytotoxicity, and better performance in BNCT. Therefore, BNCT with ANG-B may be a new option to cope with cancer.

## 2. Materials and methods

### 2.1. Cell culture

Cell lines established from human glioma (U87MG, U251, and HS683), melanoma (A375), non-small cell lung cancer (A549), and hepatocellular carcinoma (HepG2) were obtained from the National Infrastructure of Cell Line Resource in China, authenticated, and cultured in DMEM, supplemented with 10% fetal bovine serum (HyClone) and 100 IU/mL penicillin/streptomycin (all Sigma-Aldrich unless otherwise noted). Cells were routinely tested for mycoplasma using the Mycoplasma Test Kit (Cellorlab, China). All cells were maintained in a humidified incubator at 5% CO_2_ and 37°C after defrosting and cultured in the exponential growth phase before each experiment.

### 2.2. Mouse model

The glioma mouse model was established as reported recently (16). HS683 cells were transfected with luciferase-expressing vector (Lenti-UTR-Luc-Blank Vector; Promega). BALB/c nude mice were anesthetized by intraperitoneal injection of tribromoethanol (150 mg/kg) and then placed in a stereotaxic instrument. A hole was made in the mouse skull and 5 μL of HS683 cell suspension (2 × 10^7^ cells/mL) was injected into the brain using the stereotaxic instrument. The growth state of glioma tumours in mice was monitored by bioluminescent imaging (IVIS 3D Perkin Elmer). Animal experiments were performed according to the protocols approved by the Animal Use Review Board and Ethical Committee of Shenzhen Bay Laboratory (permit no. AFRQS202101).

### 2.3. Syntheses of boronated peptides

The chemical synthesis of all peptides was conducted via Fmoc solid-phase peptide synthesis using a microwave-assisted automated synthesizer (CEM Liberty Blue). Peptides were boronated with ^10^B-Boric acid (TekanBio, Shanghai, China) via the condensation reaction. Amino acid building blocks (Novabiochem), rink amide resin and coupling reagents were purchased from Annoron Biotechnology CO., LTD (Beijing, China). The peptides were purified by HPLC (Shimadzu’s LC-40) to yield products with ≥94% purity (ODS-34.6 × 250 mm, C18 column). A linear gradient of eluent B (MeCN, added 0.05% TFA) in eluent A (H_2_O, added 0.065% TFA) was applied at room temperature for purification. The purity (>95%) was confirmed by analytical RP-HPLC with UV detection at 220 nm. Molecular identity was confirmed by LC-HRMS (Vion, Waters). The boronated peptides were lyophilized and stored at −20°C until use. Amino acid sequences were validated as TFFYGGSRGKRNNFKTEEYC (ANG-B), RKKKRRQRRR (TAT-B), and MASMTGGQQMG (T7-B).

### 2.4. Thermal neutron radiation for BNCT

Thermal neutron radiation was given as described previously (16). A high-current proton beam (3.5 MeV) generated from a radio frequency quadrupole (RFQ) accelerator at the China Spallation Neutron Source was used to bombard a lithium target and produce thermal neutrons (0.5 eV-10 keV) for BNCT.

### 2.5. Boron concentration analyses

After treatment with boronated peptides or BPA (HEC Pharm, Dongguan, China), boron concentrations in human cells or mice tissues were measured by inductively coupled plasma atomic emission spectroscopy (abbr., ICP-AES; Leeman’s Prodigy 7) using published methods (16).

### 2.6. Cell proliferation and apoptosis assay

CCK8 assay was performed to measure cell proliferation after the indicated treatment conditions using the manufacturer’s protocol (Dojindo Molecular Technologies, Inc., Japan).

DNA fragmentation in apoptosis induced by BNCT was assessed using the one-step TUNEL apoptosis assay kit following the manufacturer’s protocol (Abbkine Scientific Co., Ltd., China).

### 2.7. Clonogenic cell survival assay

HS683 cells (10^6^/sample) were prepared in centrifuge tubes (1.5 mL, Eppendorf) and treated with ANG-B, TAT-B, T7-B, or BPA at various concentrations (0.0–5.0 mM) for 12 h. Epithermal neutrons were given with a total neutron flux of 3.0 ± 0.4 × 10^11^ n/cm^2^ given in 2 h. The drug-containing medium was then washed off and desirable numbers of cells in single-cell suspension were seeded into 6-well plates to grow colonies in 2 weeks. Colonies were fixed in 70% ethanol at room temperature for 10 min, and stained with 0.1% crystal violet. Colonies with more than 50 cells were scored as clonogenic survivors.

### 2.8. *In vivo* BNCT tests

During BNCT, five mice per group were injected with 2.5 mM/kg of BPA (5% fructose solution) or ANG-B solution through the tail vein. 90 min later, mice were anesthetized with an intraperitoneal injection of tribromoethanol at a dose of 150 mg/kg and then fixed in a mould for BNCT irradiation of the mouse head using a published method (16). Mice were treated with 2 kW of neutron flux for 2 h each time and four times for each mouse in 1 week, to achieve a total neutron flux of 12.5 ± 0.5 × 10^11^ n/cm^2^. Mice were anesthetized with 2% isoflurane at 25°C and imaged with PET/CT (nanoSCAN, Mediso) with 1.5 uCi/g ^18^F-FDG at 1 day before or 31 days after BNCT. Image processing and analyses were performed by AMIDE (RRID: SCR_005940), where ROI, SUV values, and tumour volume were determined.

### 2.9. Statistics

Statistical analyses were conducted using GraphPad (RRID: SCR_000306). Data from repeated experiments are presented as mean ± SD.

## 3. Results

### 3.1. Syntheses and evaluation of boronated peptides

We firstly developed three peptides into boron delivery agents. Angiopep-2 is a commonly used drug delivery ligand for directing diagnostic and therapeutic agents to the brain because it can bind to the microvascular endothelial cells and promote the BBB-crossing efficiency. Here we modified angiopep-2 with a ^10^B-4-carboxyphenylboronic acid at the N terminus and a cysteine at the C terminus to enhance the boron-10 delivery potential (Supplementary Figure S1A). The molecular mass and purity of ANG-B were evaluated using LC–MS and HPLC. According to the results of LC–MS (Supplementary Figure S1B), we identified its [M + 4H]^4+^ signal (*m/z* calc. 639.1) and [M + 3H]^3+^ signal (*m/z* calc. 851.8). Thus, the actual molecular mass of ANG-B obtained by LC–MS was 2552.4, consistent with the theoretical molecular mass of ANG-B, i.e., 2552.54. Furthermore, the chromatographic profile from HPLC showed a major peak of ANG-B with a retention time of 16.39 min, demonstrating a purity of >94% (Supplementary Figure S1C). The purified ANG-B product is a solid white powder that can be decomposed at 243.5°C.

We also constructed the arginine-rich peptides TAT-B (Supplementary Figure S2). As a short cationic peptide, TAT-B was supposedly capable of traversing the plasma membranes of eukaryotic cells. According to the LC–MS results, a peak with 1,616 ([M + 5H]^5+^, *m/z* calc. 324.2) was detected as expected. HPLC analyses showed a major peak of TAT-B with a purity of 95.1%. The purified TAT-B is a solid white powder with a melting point of 165°C. In addition, we synthesized a boron-peptide conjugate T7-B in a similar way (Supplementary Figure S3). T7-B was used as the control for comparison with ANG-B and TAT-B, because it does not contain a presumed cell-penetrating property. The molecular mass of T7-B was 1245.8 according to LC–MS with a purity >95% and a melting point at 178°C.

### 3.2. Boron delivery capability and cytotoxicity *in vitro*

**Figure 1:**
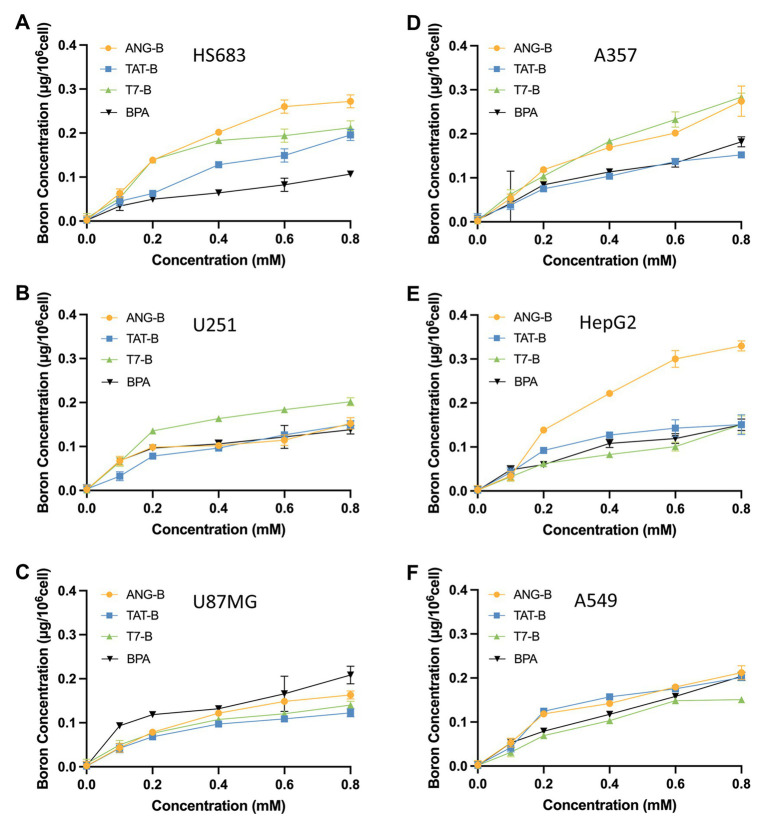
Boron concentrations in 6 human cell lines derived from glioma (U87MG, U251, and HS683), melanoma (A375), non-small cell lung cancer (A549), or hepatocellular carcinoma (HepG2). Cells were pre-exposed to 0–0.8 mM of ANG-B, TAT-B, T7-B, or BPA for 12 h. Boron concentrations were then analyzed by ICP-AES in HS683 **(A)**, U251 **(B)**, U87MG **(C)**, A375 **(D)**, HepG2 **(E)**, or A549 cells **(F)**. Mean values with standard errors are shown.

An efficient boron delivery agent should enhance boron uptake in cancer cells. Thus, we analyzed boron concentrations on 6 human cancer cell lines derived from glioma (U87MG, U251, and HS683), melanoma (A375), non-small cell lung cancer (A549), or hepatocellular carcinoma (HepG2) after treatment with various concentrations of ANG-B, TAT-B, or T7-B for 12 h. BPA was tested in parallel for comparison. Although boron concentrations were not precisely proportional to the administered doses of boron delivery agents, the gradually increasing trend suggested that cancer cells took up boron from all these agents (Figure 1). Interestingly, preference for different boron delivery agents was observed among cell types, and the three glioma cell lines showed inconsistent tastes for the tested molecules (Figures 1A–C). For example, HS683 cells preferred boronated peptides over BPA, while U87MG cells took up more BPA under the same conditions. ANG-B treatment resulted in significantly more boron uptake than BPA in HS683, A357 and HepG2 cells (*p* < 0.05; Figures 1A,D,E).

Since HS683 showed a preference in uptake of the boronated peptides, we further tested the toxicity of these drugs on this cell line. Results from both cell proliferation assay and clonogenic cell survival assay demonstrated that all the tested compounds have little cytotoxicity on cancer cells when treated alone (Supplementary Figure S4).

### 3.3. *In vitro* effects of the tested boron delivery agents in BNCT

**Figure 2:**
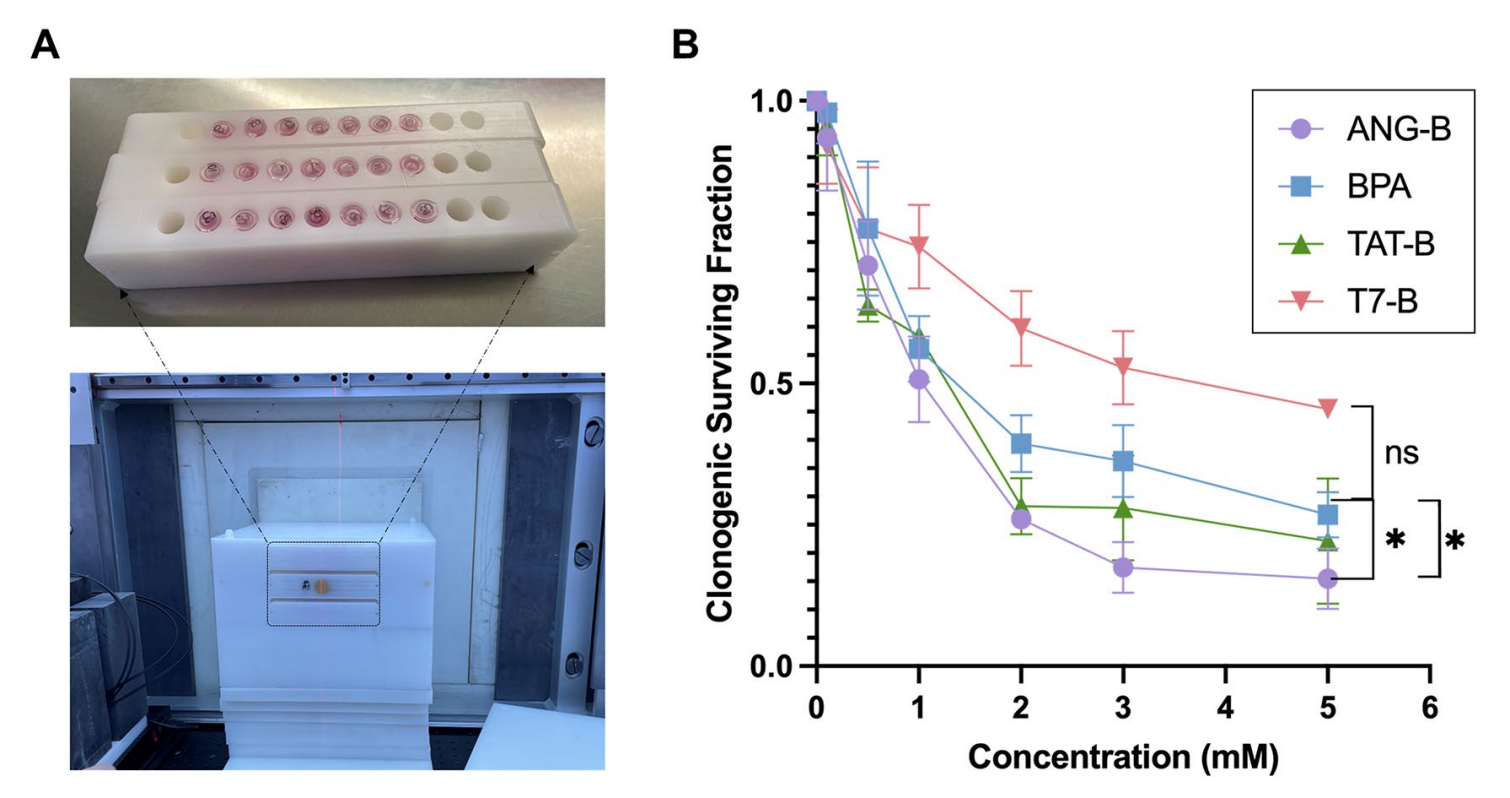
*In vitro* BNCT effects with the boron delivery agents. **(A)** Image of three Eppendorf-tube (1.5 mL) holders manufactured for thermal neutron irradiation in BNCT (upper picture). 7 of 10 holes in each holder were filled with Eppendorf-tubes containing certain numbers of cells and cell culture medium. The lower picture showed image of an immobilization mold fabricated for neutron irradiation with the tube holders (indicated by the black dashed line) in BNCT. **(B)** Clonogenic surviving fractions of HS683 cells following BNCT with 0 – 5 mM of ANG-B, TAT-B, T7-B, or BPA. Three biological repeats were performed. Two-tailed Student *t*-test; ns, not significant; ^*^ *p* < 0.05.

BNCT eliminates cancer cells by heavy charged particle rays, where reproductive cell death plays a vital role in the treatment outcome (17, 18). Here we employed the clonogenic survival assay to analyze the reproductive cell death of glioma cells after BNCT treatment *in vitro*. To guarantee the performance of BNCT *in vitro*, we designed and manufactured a tube-holding device for thermal neutron irradiation of cells (Figure 2A). BNCT with these boron delivery agents were tested on HS683 cells. Among the tested boron delivery agents, ANG-B showed the most promising effects on the glioma cells (Figure 2B). BNCT with 5 mM ANG-B caused 86.5% ± 5.3% of clonogenic cell death. By contrast, BPA at the same concentration only caused 73.3% ± 6.0% clonogenic cell death, while the number of clonogenic cell death was 77.9 ± 11.1 and 54.5% ± 1.1% for TAT-B and T7-B, respectively. Therefore, ANG-B was selected for the following *in vivo* study.

### 3.4. Boron distribution in a mouse glioma model

**Figure 3:**
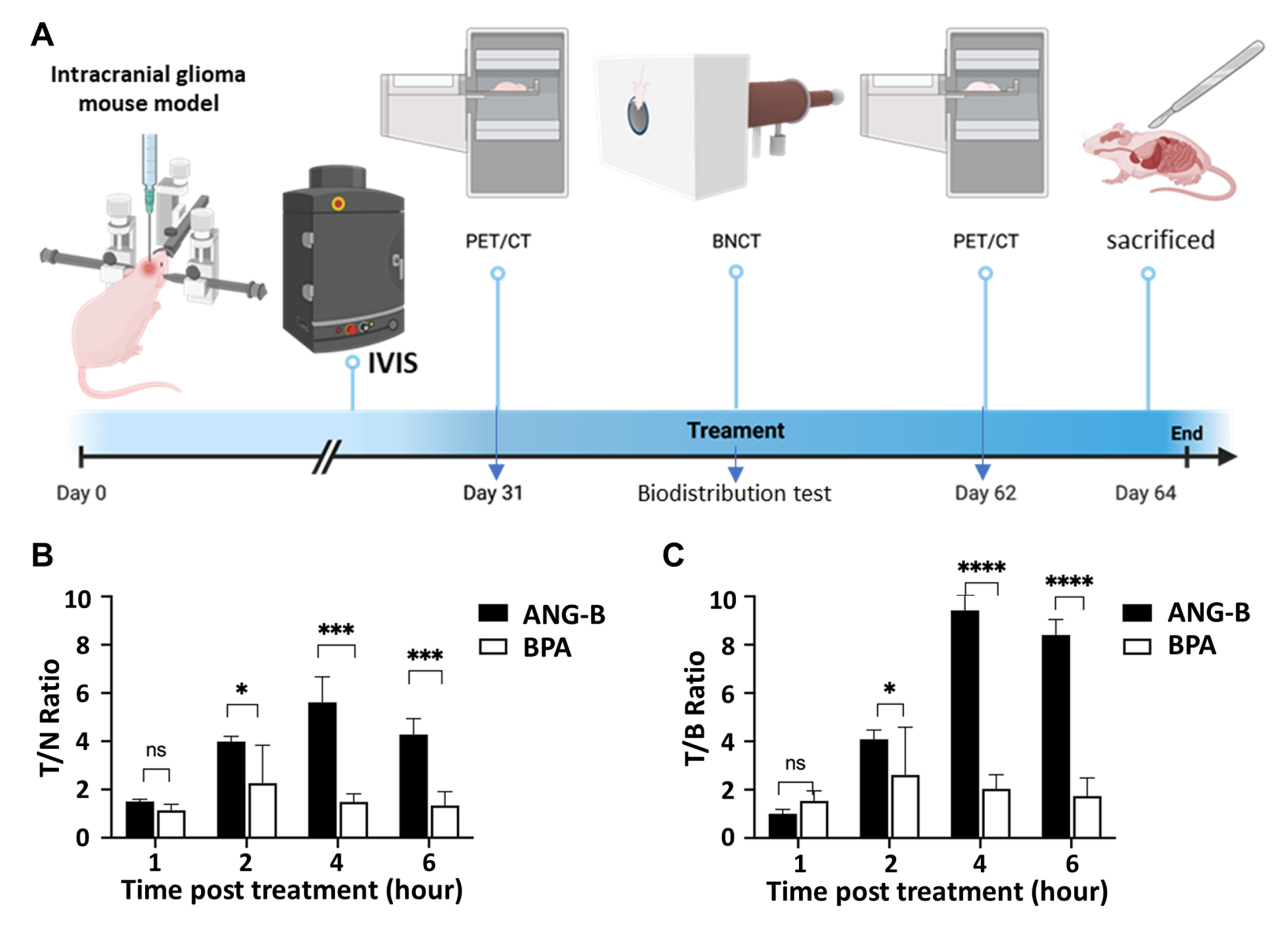
Biodistribution of boron-10 in a glioma mouse model. **(A)** Schematic illustration of the procedures for establishing an intracranial glioma mouse model, and performing IVIS monitoring, PET/CT imaging, biodistribution tests, BNCT treatment, etc. **(B)** Tumour/normal (T/N) tissue ratio after tail-vein injection of ANG-B and BPA (2.5 mM/kg) for 1, 2, 4, or 6 h. **(C)** Tumour/blood (T/B) ratio after ANG-B or BPA injection. Mean values are shown with standard errors. Three mice were analyzed for each group. Two-tailed Student *t*-test; ns, not significant; * *p* < 0.05, ^***^ *p* < 0.005, ^****^ *p* < 0.0005.

Proper microenvironmental components are critical for the treatment response of glioma (19, 20). Thus, we constructed an intracranial mouse glioma model based on HS683 cells and a research plan based on this model for testing ANG-B effects (Figure 3A). In order to monitor the intracranial tumour growth, we used luciferase-expressing cells for this mouse model (Supplementary Figure S5A). Boron biodistribution was examined at the time point for thermal neutron irradiation. 2.5 mM/kg of ANG-B or BPA (in 5% fructose solution) was injected into mice, and tissues or organs from the tumour, blood, brain, lung, liver, heart, spleen, and kidney were then collected at indicated time points for measuring boron concentrations (Supplementary Figure S5B). The glioma-targeting effects and the blood clearance of ANG-B were significantly better than that of BPA (Figures 3B,C; Supplementary Figure S5B). At 4 h after drug injection, the T/N ratio of ANG-B was 5.61 ± 1.0 and the T/B ratio was 9.42 ± 2.47.

### 3.5. BNCT effects with ANG-B or BPA *in vivo*

**Figure 4:**
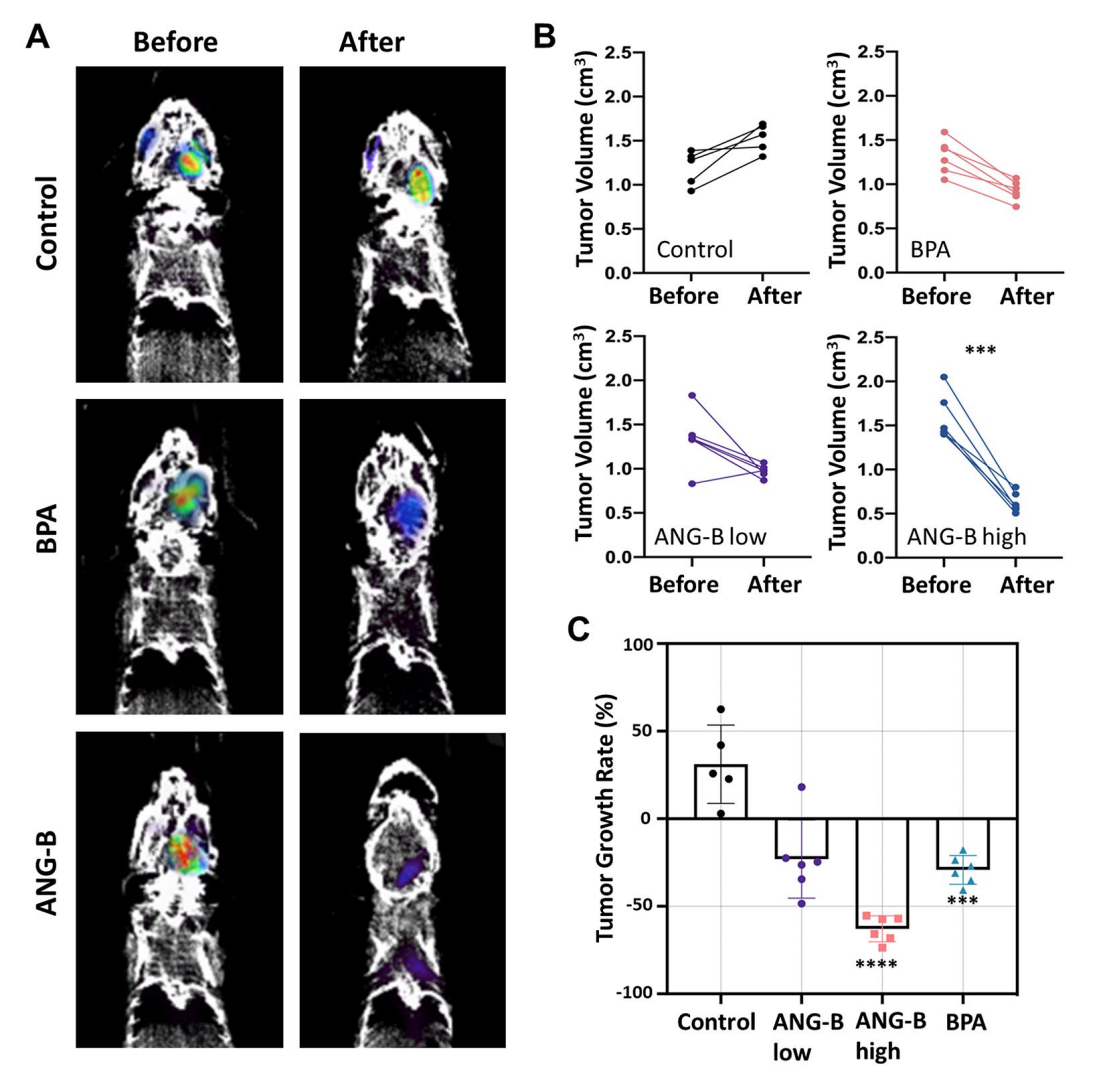
BNCT effects of ANG-B and BPA in the glioma mouse model. **(A)** Representative PET/CT images 1 day before and 31 days after BNCT treatment. **(B)** Intracranial tumour volume was measured for each mouse by PET imaging with 18F-FDG before and after BNCT treatments with BPA in 5% fructose solution (2.5 mM/kg), ANG-B solution (1 mM/kg, ANG-B low; 2.5 mM/kg, ANG-B high), or mock treatment as control. 6 mice were used in each treatment group except the control group (*n* = 5). **(C)** Tumour shrinkage rate after BNCT with ANG-B or BPA. Mann–Whitney U test and two-way ANOVA were used for statistical comparisons; ^***^p < 0.001, ^****^ *p* < 0.0005.

The glioma mouse model was further used in BCNT tests for ANG-B. The body weight of all the used mice was monitored during the whole process, where no obvious health issues with body weight loss were noticed (Supplementary Figure S6A). PET imaging is usually used to evaluate therapeutic response of glioma patients or mouse models (16, 21, 22). Therefore, we employed ^18^F-FDG-based PET/CT to estimate intracranial tumour volume for each mouse 1 day before and 31 days after BNCT treatments (Figure 4A). An additional lower concentration of ANG-B at 1 mM/kg (i.e., ANG-B low) was included for comparison.

In order to evaluate the ^18^F-FDG uptake in tumour tissues under different treatment conditions, SUV data were calculated based on a reference region (23). Compared to the control group, we found that BNCT with ANG-B low, ANG-B high, or BPA significantly decreased SUV in the tumour tissues (Supplementary Figure S6B; *p* < 0.05), suggesting that BNCT efficiently suppressed metabolic activities in tumours by eradicating cancer cells. Based on the 3D PET image data, we also estimated tumour volume before and after BNCT treatment for each mouse (Figure 4B). Consistent with the clonogenic cell survival data (Figure 2B), all the tested boron delivery agents in BNCT suppressed tumour regrowth *in vivo*, but only ANG-B at a relatively higher concentration (2.5 mM/kg) significantly decreased tumour volume at 31 days after BNCT treatment. By contrast, tumour size in the control group kept increasing. We further calculated tumour growth rate by comparing tumour size before and after BNCT, and identified that the average tumour size was reduced by 23.0, 62.9, and 29.2% for ANG-B low, ANG-B high, and BPA groups, respectively (Figure 4C). Consistent with these results, ANG-B-based BNCT gave rise to more apoptotic cells in mouse tumours at 1 day or 7 days after treatment (Supplementary Figure S6C).

## 4. Discussion

As a typical modality of binary radiation therapy, BNCT has been proposed as a potent way to cope with cancer since decades ago, although it has not been widely exploited in clinical applications to date due to the shortage of medical neutron sources and ideal boron delivery agents. Compared to traditional radiotherapy or chemotherapy, BNCT has several distinguished advantages. For example, BNCT takes advantage of the high relative biological effectiveness (RBE) of particle radiation; boron delivery agents used in BNCT usually have cancer cell selectivity, so normal tissue suffers little toxicity; conventional radiation therapy is given by as many as thirty to forty daily dose fractions, whereas BNCT only treat patients once or twice for a similar or even better tumour control probability. In a clinical study in Finland, inoperable and locally recurred head and neck squamous cell carcinoma were treated with once or twice BPA-mediated BNCT, and most patients responded (24). In a phase I/II melanoma BNCT clinical trial conducted in Argentina, 69.3% of overall response was detected regarding evaluable nodules (25). In our study with an intracranial glioma mouse model, BPA-BNCT significantly suppressed tumour regrowth (Figure 4). However, in a clinical data analysis report, the clinical outcome of BPA-BNCT arms was predicted to be inferior to the standard therapy (radiotherapy with temozolomide) (26). Thus, the current form of BNCT is unsatisfactory for glioblastoma treatment.

Boron delivery agent is one of the critical factors limiting the expansion of BNCT application in cancer treatment. An ideal boron delivery agent should be low toxicity, high tumour uptake, quick blood clearance, T/N ratio > 3, and low accumulation in healthy tissues. Unfortunately, no existing boron delivery agents can fulfil all these requirements. Historically, BPA is the most popular boron delivery agent in clinical BNCT. Cancer cells usually promote cellular amino acid transport and result in increased uptake of BPA, especially in melanoma cells (27). A study evaluated the biodistribution of BPA in melanoma patients, and the results showed that the T/N ratio was 3.40 while the skin-to-blood ratio was 1.31 (28). However, the T/N and brain-to-blood ratios in glioblastoma patients were only 1.5 to 2.4 and 0.7 to 1.0, respectively (29, 30). Our study found consistent results that T/N or T/B ratios of BPA were approximately 2 in the glioma mouse model (Figure 4). In addition, BPA selectively accumulates in dividing cells but not in quiescent tumour cells (27). Thus, cancer recurrence may occur after BPA-based BNCT, even if the initial therapeutic response is good. Consequently, novel boron delivery agent with better performance in targeted cancer delivery is still an unmet need.

In this study, we developed novel boron-peptide conjugates for BNCT. We considered cell-penetrating property when designing these peptides (11). As a class of peptides usually consisting of 5–30 amino acids with a net positive charge, CPPs can carry therapeutic molecules across cell membranes (10). We selected angiopep-2 because it shows excellent transcytosis ability and brain penetration capability (15). In contrast to the common strategy with cargo for therapeutic agent delivery, we directly conjugated angiopep-2 with a boron-10 at the N-terminus. We tested ANG-B in 6 cancer cell lines from different tumour entities. ANG-B performed better than BPA regarding cellular uptake *in vitro* or biodistribution *in vivo*, especially for HS683 glioma cell models. In addition, we noticed that cell lines have different preference in boron delivery agents, suggesting that, like other treatment modalities, personalized BNCT is required for optimal effects.

Based on the experimental results, we concluded that peptides with cell-penetrating ability are exploitable tools for targeted delivery of boron-10 in cancer treatment with BNCT. The novel BNCT agent ANG-B can penetrate malignant cells and accumulate in the tumour. By testing on an intracranial glioma mouse model, we confirmed that boron-10 was efficiently delivered to the tumour by ANG-B and resulted in better tumour growth control than BPA. Therefore, ANG-B may improve BNCT performance in glioma treatment. Further testing of ANG-B in clinical settings is warranted.

## Data availability statement

The original contributions presented in the study are included in the article/Supplementary material, further inquiries can be directed to the corresponding authors.

## Ethics statement

The animal study was performed following the approved protocol by the Animal Use Review Board and Ethical Committee in Shenzhen Bay Laboratory (Permit No. AFRQS202101).

## Author contributions

QL and QR: conceptualization and supervision. JX, JL, NZ, WH, YL, LM, and JT: methodology and data curation. JX, QL and LM: investigation. QL, QR, and TL: resources. QL and LM: writing—original draft preparation. JX, QL, QR, and LM: writing—review and editing. All authors contributed to the article and approved the submitted version.

## Funding

This research was funded by the third foster plan in 2019 “Molecular Imaging Probe Preparation and Characterization of Key Technologies and Equipment” (ZDKJ20190305003) for the development of key technologies and equipment in major science and technology infrastructure in Shenzhen (QR and QL), the open funds of Guangdong Province Key Lab of Biomedical Imaging (GPKLBI202109; LM and QL), the Program for Guangdong Introducing Innovative and Enterpreneurial Teams (2017ZT07S225; TL and JT), and the Shenzhen Science and Technology Program (1210318663; QR).

## Conflict of interest

The authors declare that the research was conducted in the absence of any commercial or financial relationships that could be construed as a potential conflict of interest.

## Publisher’s note

All claims expressed in this article are solely those of the authors and do not necessarily represent those of their affiliated organizations, or those of the publisher, the editors and the reviewers. Any product that may be evaluated in this article, or claim that may be made by its manufacturer, is not guaranteed or endorsed by the publisher.
