# Supplementary material for: Boron-peptide conjugates with angiopep-2 for boron neutron capture therapy
Source: Front Med (Lausanne). 2023 Jun 1;10:1199881. doi: 10.3389/fmed.2023.1199881 (PMC10267362; doi:10.3389/fmed.2023.1199881)
Supplement: Supplementary file 1 [file Data_Sheet_1.docx]

Supplementary Material

Boron-Peptide Conjugates with Angiopep-2 for Boron Neutron Capture Therapy

Jing Xiang ^1†^, Lin Ma ^2†^, Jianfei Tong ^3,4^, Nan Zuo ^5,6^, Weitao Hu ^7^, Yupeng Luo ^8^, Junqi Liu ^9^, Tianjiao Liang ^3,4^, Qiushi Ren ^1,5*^, Qi Liu ^10,5*^

*** Correspondence:** Qi Liu, liu_qi@szu.edu.cn; or Qiushi Ren, [renqs@szbl.ac.cn](mailto:renqs@szbl.ac.cn)

#
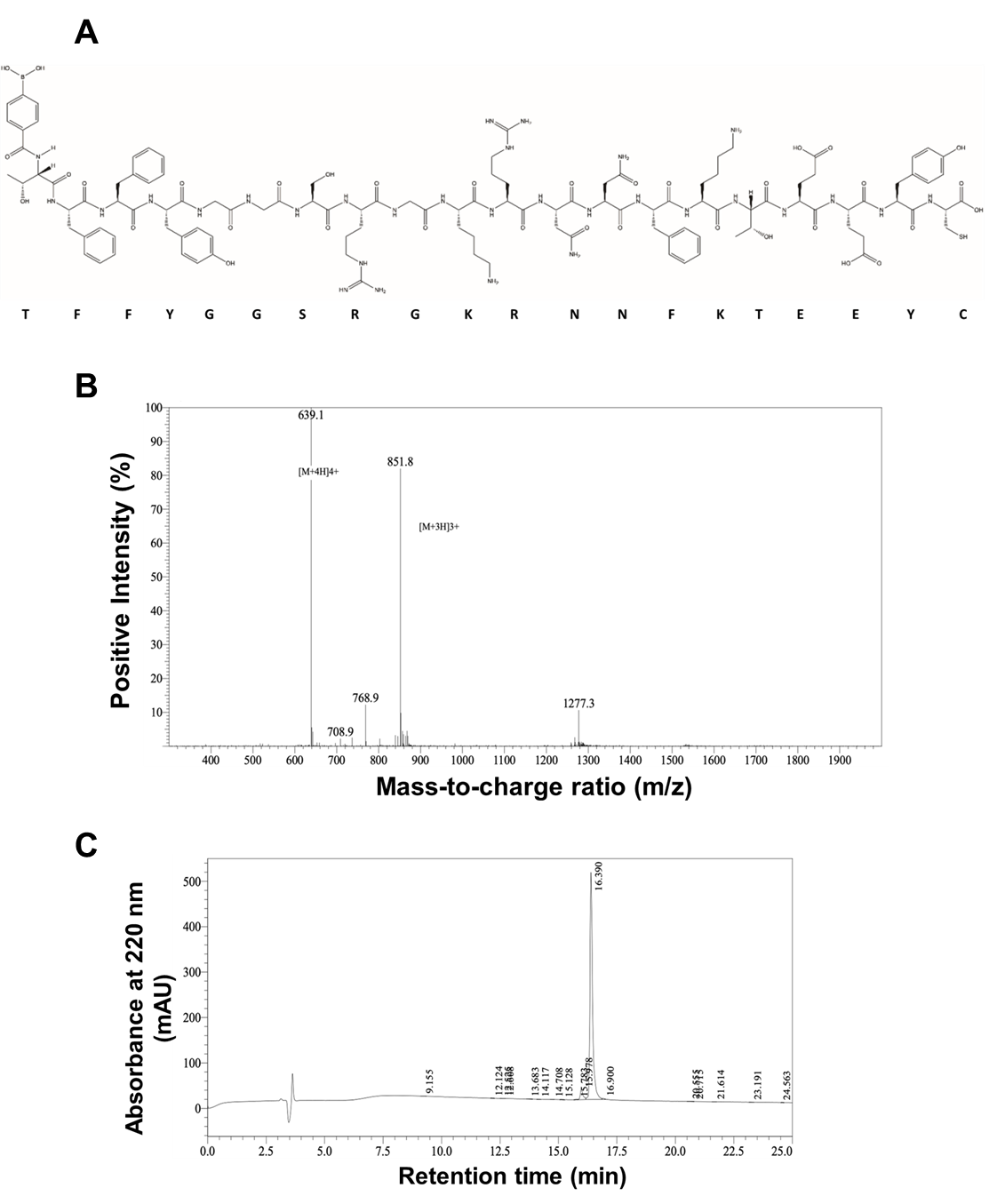
Supplementary Figures

**Supplementary Figure 1.** Chemical structures and properties of ANG-B produced following the solid-phase peptide synthesis protocol. **(A)**, The peptide (TFFYGGSRGKRNNFKTEEYC) was modified with a ^10^B-4-Carboxyphenylboronic acid at the N terminus to synthesize ANG-B; **(B)**, The positive intensity of ionized ANG-B according to their mass-to-charge ratio analyzed by mass spectrometry; **(C)** HPLC analysis of components in the synthesized ANG-B products.


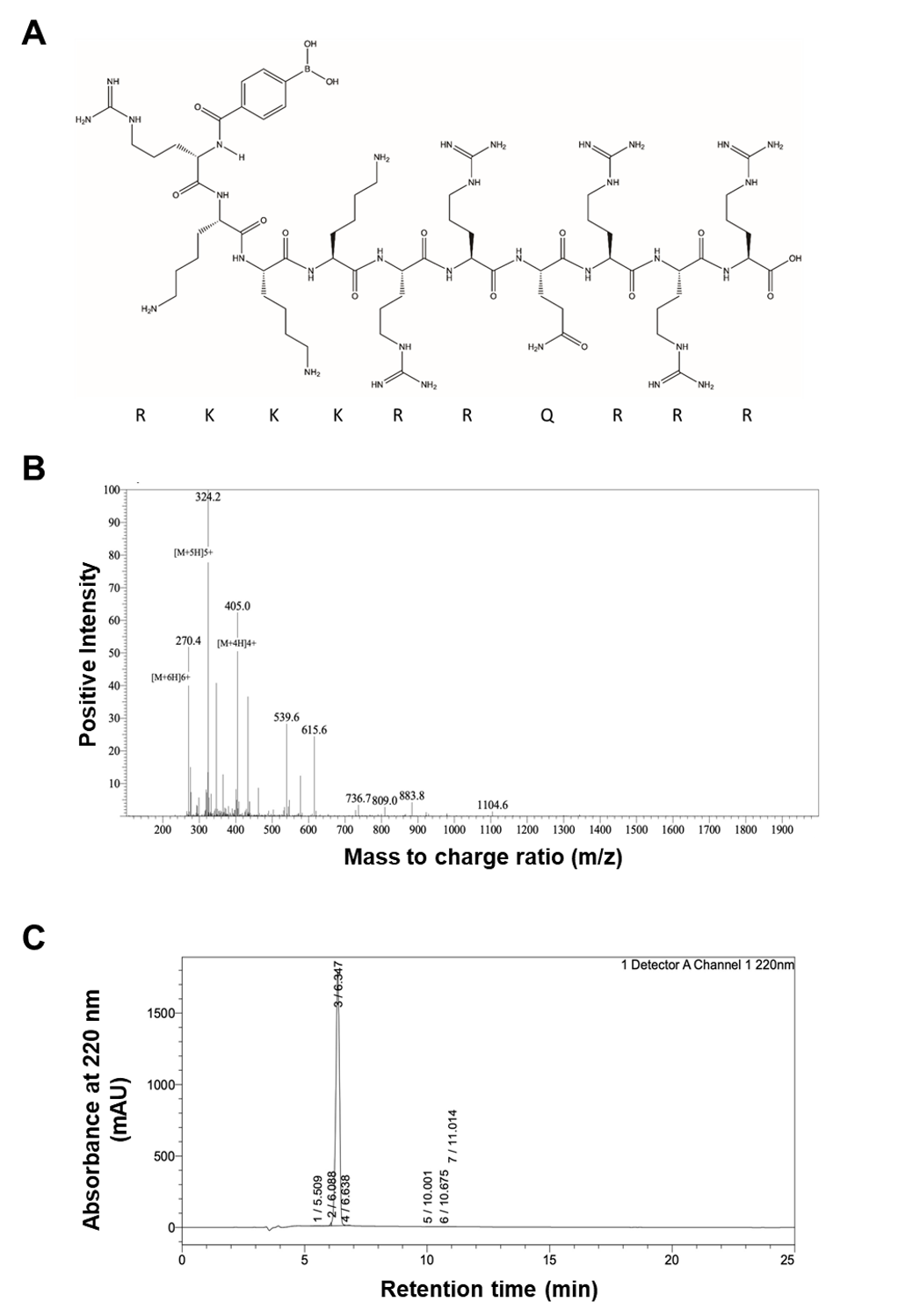
**Supplementary Figure 2.** Chemical structures and properties of TAT-B produced following the solid-phase peptide synthesis protocol. **(A)**, The peptide (RKKKRRQRRR) was modified with a 10B-4-Carboxyphenylboronic acid at the N terminus to synthesize TAT-B; **(B)**, The positive intensity of ionized TAT-B according to their mass-to-charge ratio analyzed by mass spectrometry; **(C)** HPLC analysis of components in the synthesized TAT-B products.


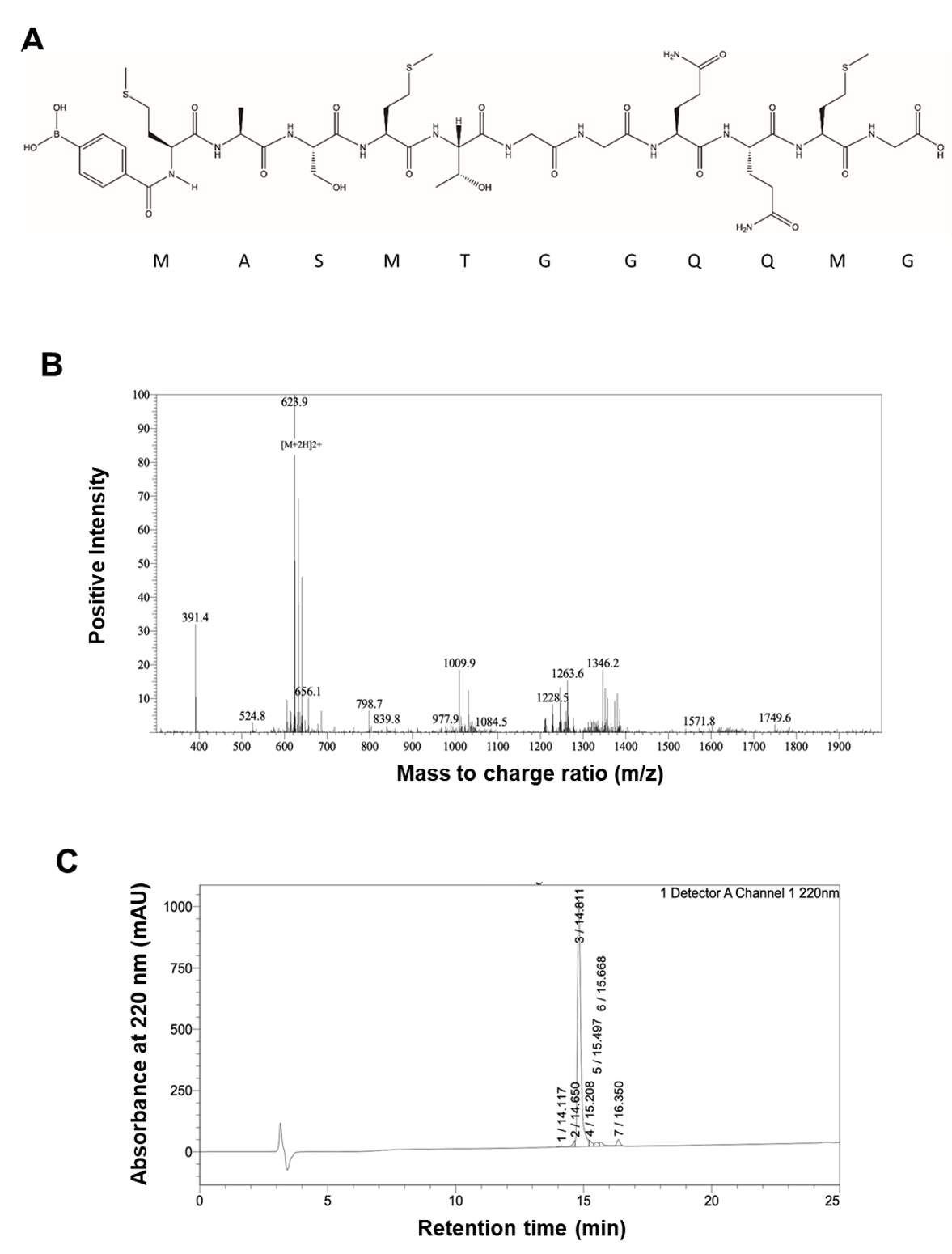


**Supplementary Figure 3.** Chemical structures and properties of T7-B produced following the solid-phase peptide synthesis protocol. **(A)**, The peptide (MASMTGGQQMG) was modified with a ^10^B-4-Carboxyphenylboronic acid at the N terminus to synthesize T7-B; **(B)**, The positive intensity of ionized T7-B according to their mass-to-charge ratio analyzed by mass spectrometry; **(C)** HPLC analysis of components in the synthesized T7-B products.


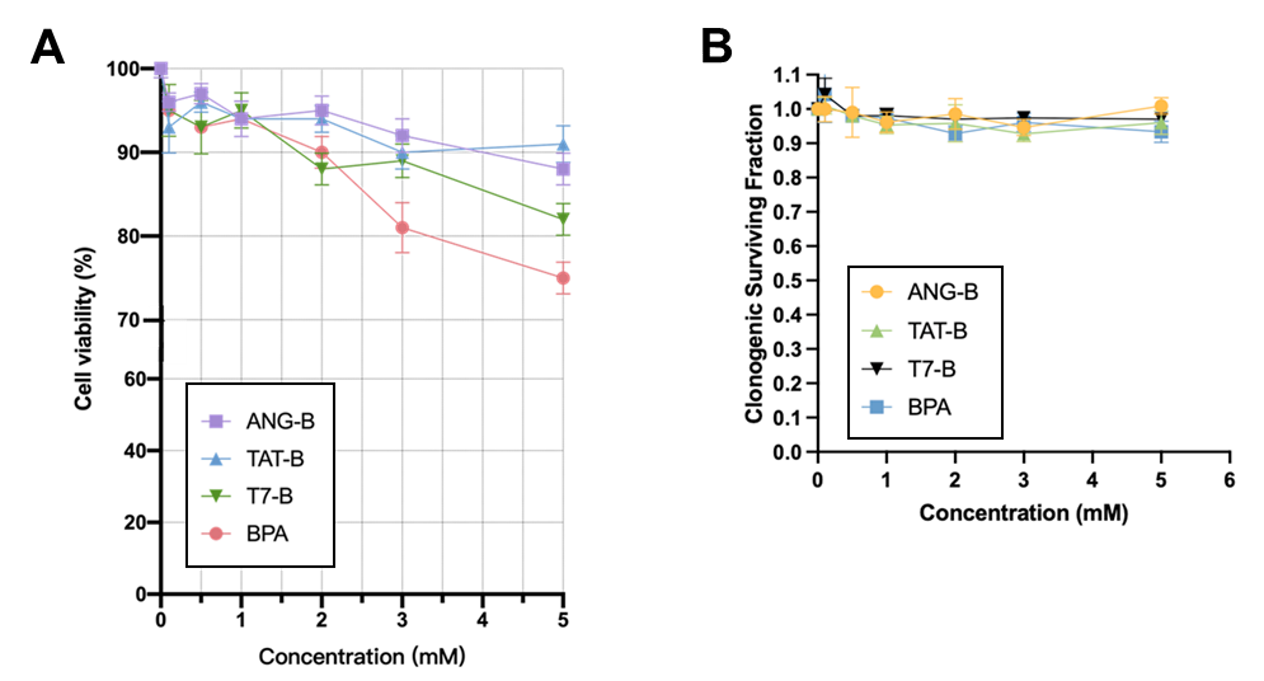


**Supplementary Figure 4.** Cytotoxicity of ANG-B, TAT-B, T7-B, and BPA when 0 - 5.0 mM drugs were administered on HS683 cells. **(A)** Percent of survived cell was measured by the CCK8 assay. **(B)** Clonogenic surviving fractions of HS683 cells treated with various concentrations of the tested boron delivery agents.


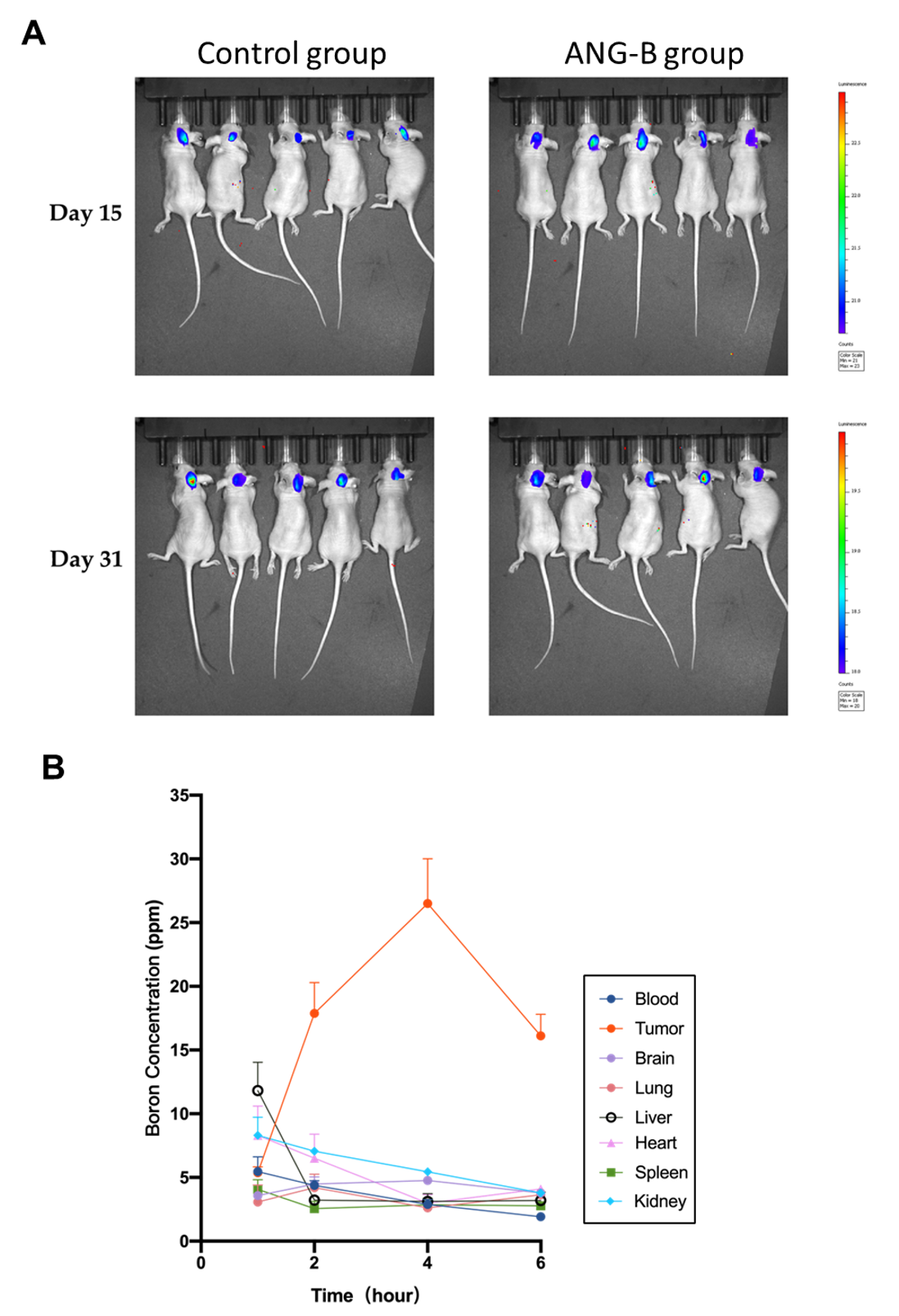


**Supplementary Figure 5.** In vivo distribution of boron-10 delivered by ANG-B in an intracranial mouse glioma model. **(A)** IVIS imaging of the mouse model at day 15 or day 31 after HS683 cell transplantation. **(B)** Boron concentrations in the tumor, blood, heart, lung, liver, spleen, and kidney after ANG-B injection (2.5mM/kg).


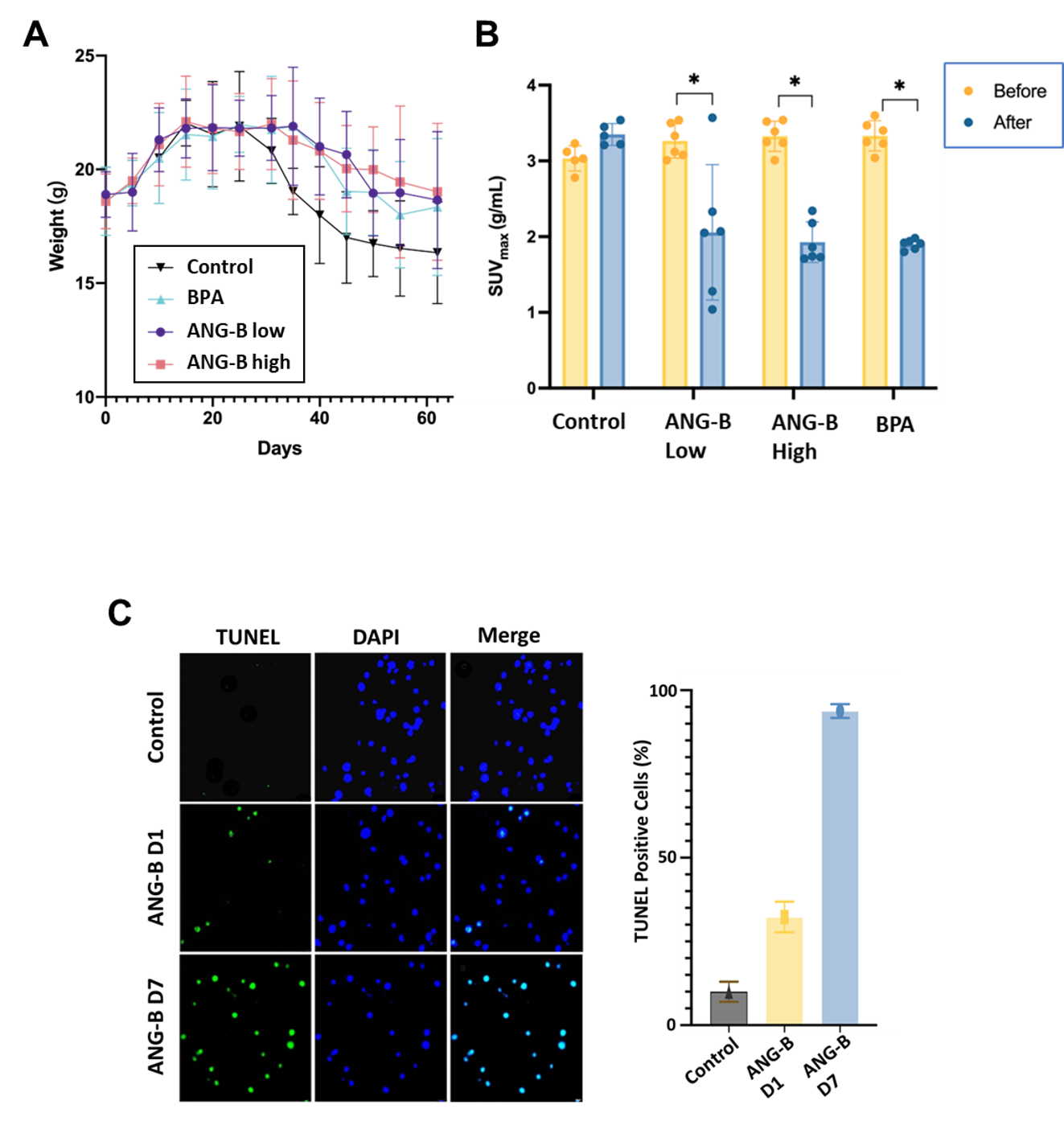


**Supplementary Figure 6.** In vivo effects of ANG-B based BNCT. **(A)** Body weight of glioma mouse model after treatment. **(B)** SUVmax analyses (indicating of FDG uptake) of glioma tumors in PET/CT imaging before and after BNCT treatment. **(C)** Apoptotic cells in tumor at 1 day or 7days after ANG-B based BNCT treatment as measured by TUNEL assay. Mann–Whitney U test; **p* < 0.05.
